# Dopaminergic Identity of SH-SY5Y Cells Across Differentiation Protocols in Parkinson’s Disease Research: A Systematic Review

**DOI:** 10.3390/ijms27083355

**Published:** 2026-04-08

**Authors:** Osvaldo Artimagnella, Alessia Floramo, Giovanni Luca Cipriano, Veronica Argento, Maria Lui

**Affiliations:** IRCCS Centro Neurolesi “Bonino-Pulejo”, Via Provinciale Palermo, Contrada Casazza, 98124 Messina, Italyalessia.floramo@irccsme.it (A.F.); veronica.argento@irccsme.it (V.A.); maria.lui@irccsme.it (M.L.)

**Keywords:** SH-SY5Y, Parkinson’s disease, dopaminergic markers, differentiation protocols, retinoic acid, TPA, BDNF

## Abstract

The SH-SY5Y cell line is widely used as an in vitro model for pharmacological and molecular investigations of Parkinson’s disease (PD). The use of SH-SY5Y cells in PD research critically relies on their ability to differentiate into a mature, post-mitotic, dopaminergic (DAergic) neuronal phenotype. However, SH-SY5Y cells are inherently heterogeneous since they are firstly catecholaminergic cells and may express diverse phenotypic markers besides the DAergic ones. These properties seem to be determined by the differentiation protocol that is employed, thus meaning it is crucial to obtain proper cell types. This systematic review aims to discuss the main differentiation protocols used in PD research over the last 30 years. They include inducers such as retinoic acid (RA), the phorbol ester TPA, and the BDNF. Among the 514 studies that were screened, 249 employed these inducers. Then, we quantitatively report the ability of these protocols to differentiate SH-SY5Y cells in mature DAergic neurons, evaluating morphology, differentiation markers, and DAergic markers among the studies that specifically compared differentiated to undifferentiated SH-SY5Y cells (61 studies over 249). As our research shows, despite the highest usage of the RA differentiation protocol, the combination of RA with the BDNF inducer seems to increase the expression and the acquisition of a DAergic phenotype. Nevertheless, during this analysis, some limitations emerged, highlighting the intrinsic phenotypic heterogeneity of these cells, thereby limiting their suitability according to the specific biological question under investigation. A deep investigation into the literature about the molecular phenotypic features of differentiated SH-SY5Y cells may eventually help us to understand the advantages and disadvantages of each protocol that was employed, and adequately set experiments around the PD research.

## 1. Introduction

Parkinson’s disease (PD) is among the most prevalent neurodegenerative disorders, primarily affecting dopaminergic (DAergic) neurons within the substantia nigra. The pathology is characterized by the presence of α-Synuclein protein aggregates, which are the primary constituent of Lewy bodies, leading to dopamine (DA) depletion and subsequent neuronal death [1]. As a neurotransmitter, DA is essential for regulating various biological functions; it plays a critical role in motor control via the nigrostriatal pathway, modulating basal ganglia circuitry and motor performance [2]. Furthermore, DA mediates the reward system [3], pleasure perception [4], motivation [5], and mood regulation [6]. Consequently, DA deficiency results in significant motor and cognitive dysfunctions that are characteristic of PD.

To elucidate the pathophysiological mechanisms underlying PD and develop effective therapeutic strategies, the utilization of robust in vivo and in vitro models is imperative. Preclinical research relies heavily on in vivo rodent models [7,8], followed by simpler organisms such as *Drosophila melanogaster* and *Caenorhabditis elegans* [9]. Conversely, in vitro cellular models serve as indispensable tools, offering advantages in terms of cost-effectiveness, reduced temporal requirements, and superior experimental control compared to animal models [10].

In vitro models are the preferred choice for investigating the intricate pathogenic mechanisms and the molecular effects of neuroprotective agents. The primary neuronal cultures derived from rodent brain tissue provide a suitable in vitro model to accurately mimic DAergic function. However, their sensitivity and rapid degradation under ex vivo conditions limit their long-term experimental utility [11]. Recent advancements have been bolstered by induced pluripotent stem cells (iPSCs) derived from PD patients. These cells can be differentiated into DAergic neurons to model both familial and idiopathic forms of the disease while preserving the patient’s specific genetic background [12]. Furthermore, the development of 3D midbrain organoids represents a groundbreaking approach, as these models mimic complex neuronal networks and replicate key PD phenotypes, such as progressive DAergic loss and neurodevelopmental alterations. These three-dimensional systems constitute a vital bridge between traditional 2D cultures and in vivo animal models [13].

Despite their high fidelity, these advanced models are expensive, time-consuming, and necessitate specialized expertise. Moreover, iPSC- and 3D organoid-based models exhibit substantial heterogeneity due to donor-specific genetic background, variations introduced during reprogramming and culture conditions. Together, these factors limit protocol standardization, reduce reproducibility across laboratories, and represent a significant challenge for clinical translation [14,15].

In this context, human immortalized cell lines represent a practical compromise for pharmacological and mechanistic investigations. The SH-SY5Y human neuroblastoma cell line is one of the most widely utilized models in PD research due to its catecholaminergic properties and capacity for differentiation into neuron-like cells using specific protocols. However, a significant controversy persists in the literature regarding the efficacy of several differentiation protocols in upregulating DAergic markers [16,17].

This systematic review aims to provide a quantitative analysis of the literature concerning the use of differentiated SH-SY5Y cells in PD research, evaluating their molecular phenotypic profiles. We firstly report the cellular properties of the SH-SY5Y cell line, the methods to induce a PD model, and the primary differentiation protocols employed in the literature, including those involving retinoic acid (RA), phorbol ester 12-O-tetradecanoylphorbol-13-acetate (TPA), or the brain-derived neurotrophic factor (BDNF). Furthermore, we examine the capacity of these protocols to induce a mature DAergic phenotype by assessing the neuritic morphology and the expression of neuronal and DAergic markers. Specifically, we report trends of frequency usage over years across differentiation protocols; we perform an evaluation analysis of the main markers that were regulated during differentiation; and we present works that directly compare two or more protocols. Finally, we discuss the main findings obtained from the systematic analysis, concluding with a work hypothesis and future perspectives. A comprehensive exploration of the molecular phenotypic characteristics of differentiated SH-SY5Y cells is essential for understanding the relative advantages and limitations of each protocol, ultimately allowing researchers to align their experimental design with specific biological questions in PD research.

## 2. Methodology

### 2.1. The Literature Search, Study Selection, and Data Analysis

**Figure 1:**
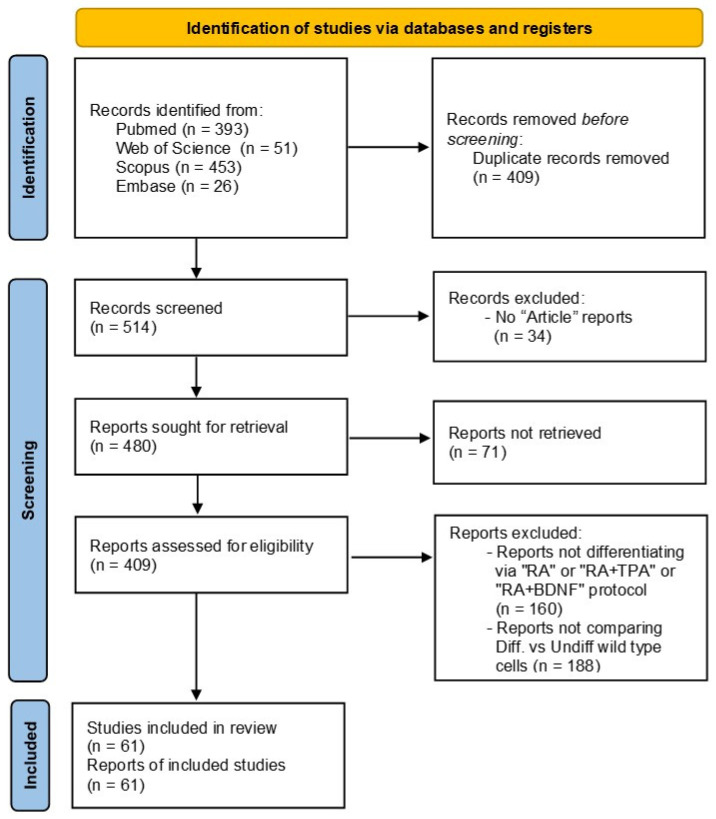
A PRISMA flow diagram detailing the study selection process of this systematic review. It includes the identification and screening steps to reach the included studies, with the criteria of exclusion. It was adapted starting from the PRISMA flow diagram template.

This systematic review was conducted according to the preferred reporting items for systematic reviews and meta-analyses (PRISMA) guidelines [18]. The review protocol was not registered, and the study selection process is detailed in the PRISMA flow diagram (Figure 1). Specifically, we retrieved from PubMed, Scopus, Web of Science and Embase databases every article about the usage of differentiated SH-SY5Y cells in PD research, using the following query: “(SH-SY5Y OR SHSY5Y) AND Parkinson AND (Differentiation OR Differentiate OR Differentiated)”. The search was run over a period of 30 years, from 1995 to 2024, collecting a total of 923 articles. 

Before screening, we removed the duplicated records (n = 409). Then, the remaining 514 reports were screened, removing those that were not annotated as “Article” type (n = 34) and that were not accessible to our institution (n = 71). Post hoc filtering was applied to the 409 studies retained, dividing them into three main groups according to the differentiation protocol that was employed: (1) “RA” refers to studies which use an RA inducer; (2) “RA+TPA” refers to studies using a combination of both RA and TPA inducers since we only found four papers that administered the TPA alone, so to improve readability we decided to include them in the group “RA+TPA”; and (3) “RA+BDNF” refers to the studies employing the RA inducer together with the BDNF. It is worth noting that our query search also included articles that did not report any of these differentiation protocols (a total of 160), which were therefore filtered out. The remaining studies amounted to 249 in total.

Next, to evaluate the effects of differentiation protocols on SH-SY5Y cells, we selected only studies that compared undifferentiated SH-SY5Y cells and differentiated ones, excluding all studies lacking a direct comparative analysis between the two conditions. Therefore, 61 works satisfied this condition and were thoroughly screened to ultimately collect information about the differences in morphology, the differentiation markers and the DAergic markers. For this evaluation, we considered: microscopy data; gene expression via quantitative PCR; immuno-related methods (such as Western blots and flowcytometry); and omics approaches, including transcriptomics (e.g., via sequencing and microarray); and proteomics (via mass spectrometry). Since some studies included more than one protocol of differentiation, in this review, we considered the number of protocols examined, which was a total of 72. To perform analyses and to build tables and graphs, we considered those markers that were reported at least twice among these protocols. For each marker, we noted whether its expression was significantly upregulated (“UP”), downregulated (“DOWN”), or not regulated (“NS”), comparing the differentiated and undifferentiated SH-SY5Y cells. Importantly, we set the entire analysis, assuming an increased expression of markers (or decreased expression only for proliferative markers), in differentiated cells rather than undifferentiated ones, while considering all the protocols not able to induce a significant change in marker expression between the two conditions as not effective. Finally, some works compared two or more protocols that were deeply analyzed. All the primary data generated during this review are included in Table S1.

### 2.2. Methodological and Data Limitations

Here, we will highlight some limitations of this review. First, we exclusively included studies whose main subject of investigation is PD and that specifically investigate and primarily focus on DAergic evaluations, without addressing other phenotypic markers. To address this limitation, we extended the literature research to include additional works and reviews describing the association between the SH-SY5Y cells and other phenotypes, such as cholinergic and adrenergic ones. Similarly, this analysis could be further expanded by also considering in vitro studies with SH-SY5Y cells as models of other disorders, such as Alzheimer’s disease (AD) [19,20,21].

The significant difference in numerosity among the three differentiation protocols may cause a potential misrepresentation of the results. Moreover, none of the studies included in this review directly made a comparison between the TPA- and BDNF-based differentiation protocols, thereby limiting our understanding of the results and impacting the robustness of the data interpretation.

Additionally, because the SH-SY5Y cells are of neuroblastoma (neoplastic) origin, the commercially available batches display substantial heterogeneity that depends on clone, supplier, basal medium, serum supplement, and other culture conditions [22,23]. Differences in the maintenance and differentiation protocols may alter the morphology and differentiation capacity, compromising reproducibility [24]. Some of the 61 primary studies (Table S1) did not report differentiation details, such as inducer concentration, serum levels, or protocol duration, which hindered the precise protocol comparisons and standardization.

Lastly, the final limitation concerns the insufficient reporting of the cell culture quality control practices. Among them, only 6 studies explicitly reported the STR-based cell line authentication or mycoplasma testing, and only 15/61 studies specified the passage range used for the SH-SY5Y cultures (usually below passage 20). Since prolonged passaging may alter the differentiation capacity and phenotypic stability of the SH-SY5Y cells [24], the lack of this information may represent an additional source of methodological variability across the studies.

## 3. SH-SY5Y Cells: Characteristics, Differentiation Protocols, and PD Model Induction

### 3.1. Cellular and Phenotypic Properties of the SH-SY5Y Cell Line

**Figure 2:**
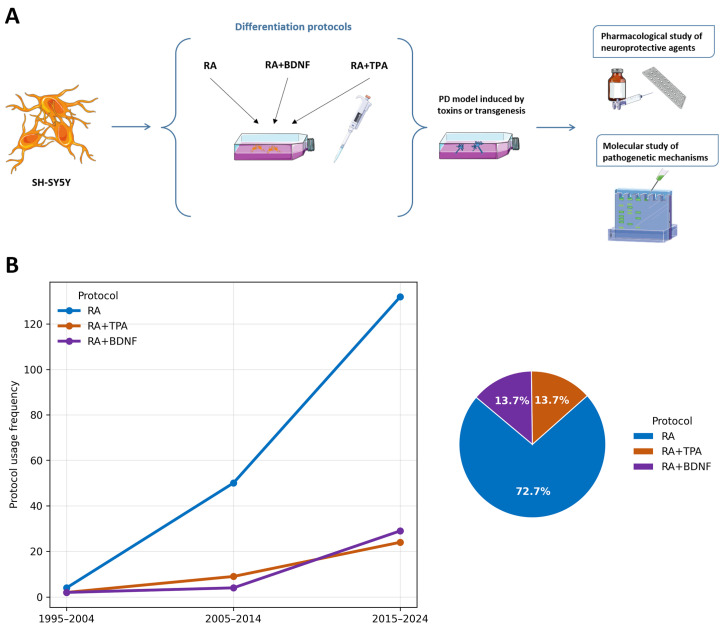
Differentiation protocols in PD research. (**A**) A graphical representation of the in vitro SH-SY5Y cell line culture to model and investigate PD. (**B**) The use of differentiation protocols in PD research over the past 30 years. On the left, a line chart shows the percentage trend in the use of the main differentiation protocols (RA, RA+TPA, and RA+BDNF) divided into three time intervals: 1995–2004, 2005–2014, and 2015–2024. On the right, the pie chart reports the overall percentages of the three differentiation protocols that were used over the past 30 years. These observations support what has already been reported in the previous literature [22,27].

The SH-SY5Y neuroblastoma cell line is extensively employed as an in vitro model for neurodegenerative disorders, such as AD and PD. It serves as a fundamental tool for investigating pathogenetic mechanisms and evaluating the neurotoxicity or neuroprotective potential of various pharmacological treatments (Figure 2A). Phenotypically, this cell line exhibits significant heterogeneity; it is characterized by both adherent and floating cells, with the population comprising epithelial-like (S-type) and neuroblast-like (N-type) morphologies [25]. Furthermore, N-type cells are predominantly catecholaminergic, expressing both tyrosine hydroxylase (TH) and dopamine-β-hydroxylase (DβH) [26]. TH serves as the rate-limiting enzyme in catecholamine biosynthesis, catalyzing the conversion of L-tyrosine to L-3,4-dihydroxyphenylalanine (L-DOPA). Subsequently, DβH facilitates the conversion of DA into norepinephrine (noradrenaline, NA), highlighting the intrinsic capacity of these cells to synthesize both neurotransmitters.

The ability to differentiate the SH-SY5Y cells into a mature, neuron-like phenotype using dedicated inducers has provided numerous advantages to the neuroscience field. These benefits include the capacity for extensive culture expansion prior to differentiation, cost-effectiveness, and ease of maintenance. Furthermore, as human-derived cells, they express human-specific isoforms and circumvent the ethical concerns associated with in vivo models. Collectively, these attributes render the SH-SY5Y cells particularly suitable for investigating PD-related biological processes, such as neurotoxicity, α-Synuclein aggregation, and large-scale drug screening, offering a practical alternative to primary neurons and iPSCs [27].

In the undifferentiated condition, the SH-SY5Y cells show characteristics similar to those of neoplastic cells, with high proliferative capacity, rounded morphology and small neurite extensions [22]. In contrast, significant changes have been observed during the differentiation process: the cells develop an extensive neuritic network, which is associated with a reduction in proliferation that is typical of an advanced state of maturation; they adopt a pyramidal shape [28,29]; and finally, they increase the electrical excitability of the plasma membrane [30]. These changes make these cells very similar to primary neurons [27].

It is important to highlight that differentiated SH-SY5Y cells exhibit phenotypic plasticity, meaning they are able to acquire DAergic, cholinergic, and adrenergic traits, depending on media conditions [19,31]. A variety of methods exist for the induction of their differentiation. Nevertheless, there are some limitations that researchers need to consider, such as the difficulty of obtaining a uniform and stable phenotype and the likelihood of not reaching a fully mature state of differentiation, with the consequent risk of using an in vitro model that does not fully reflect the functional properties of adult human neurons. In this regard, the standardization of differentiation protocols is essential to improve the reproducibility and reliability of the studies [31,32].

### 3.2. Primary Differentiation Protocols Employed with SH-SY5Y Cell Line in PD Research

The three main inducer molecules used to differentiate the SH-SY5Y cells in PD research are RA, TPA, and BDNF, which were applied alone or in combination according to three distinct protocols: RA alone, RA with TPA (RA+TPA), or RA with BDNF (RA+BDNF), as highlighted in Figure 2A. Over the last 30 years, we identified 249 studies (Table S1) reporting the use of in vitro PD models based on differentiated SH-SY5Y cells. These works investigated the cellular and molecular mechanisms of PD as well as the effects of the pharmacological treatments in the neuroprotection context. We graphed the percentage of each protocol usage in three time intervals (1995−2004, 2005−2014, and 2015−2024), showing that, above all, the RA protocol emerged as the most widely used to differentiate SH-SY5Y cells, with a percentage of 72.7% (Figure 2B). This trend was ultimately confirmed over the last ten years, during which the number of studies using the RA protocol increased significantly faster than those employing the RA+TPA or RA+BDNF protocols, which, in contrast, slowly increased over the years. The counts were based on the number of protocols rather than the number of included studies, as some studies examined multiple protocols.

RA is a vitamin A derivative that induces cell differentiation by regulating gene expression through retinoic acid receptors and retinoid X receptors [33]. RA is used at concentrations ranging from 1 μM [34] to 20 μM [35], with 10 μM being the most commonly employed concentration [17]. Similarly, the time of exposure widely ranged from 48 h [36] to 15 days [37]. Encinas and their colleagues [25] reported that upon differentiation with RA 10 μM for 5 days, the N-type cells acquire neuron-like characteristics, whereas the S-type ones exhibit signs of toxicity. In the former, cell survival is promoted by the activation of the PI3K/AKT pathway and the increased expression of the anti-apoptotic protein Bcl-2 [38]. However, they documented that the percentage of the S-type cells in the culture increased after 10 days of RA treatment, suggesting that their proliferation is promoted with longer treatment. Furthermore, in a study by Marlet et al. [39], a preliminary treatment with bone morphogenetic protein 4 (BMP4) was introduced, which promoted the proliferation of the N-type cells over the S-type cells. This approach should allow the generation of a more homogeneous cell population that would be better suited to the subsequent differentiation induced by RA and TPA. However, other studies reported that the RA-differentiated SH-SY5Y cells expressed DAergic and cholinergic markers and produced small quantities of noradrenaline as well, further complicating the interpretation of their neurochemical profile [19,21,31,40].

TPA is a phorbol ester that allows the acquisition of neuronal characteristics via the activation of protein kinase C and the modulation of various intracellular pathways [41]. Some studies employing TPA alone reported that the TPA-differentiated SH-SY5Y cells increased the cellular noradrenaline content, suggesting a predominantly adrenergic phenotype [42,43,44,45]. In the PD context, the most used protocol involves the sequential use of RA for about 5 days, followed by TPA at 80 nM for a further few days [27,46]. This combination would help the SH-SY5Y cells to differentiate towards DAergic phenotypes.

Finally, the BDNF is a neurotrophic polypeptide that plays a fundamental role not only in maintaining neuronal cell survival but also in maturation and promoting both neurite extension and the formation of new synapses. It acts through the activation of the PKB/CREB pathway, as well as the PI3K/AKT/MAPK pathway [47,48,49]. Its usage is generally reported in combination with RA, both sequential and concomitant modes of administration, at a concentration of 50 ng/mL. The use of this protocol appears to induce characteristics that are typical of a mature neuronal phenotype, although it does not ensure specific DAergic line selection [50]. Therefore, RA+BDNF-differentiated cells were reported to express markers either belonging to noradrenergic [25], DAergic [51], or cholinergic [52] phenotypes.

Regarding the other medium components, the serum is usually kept at low concentration levels, ranging from 10% to 0%, or it is gradually reduced, which is independent of the specific protocol. This helps promote differentiation and inhibit proliferation. Moreover, some studies reported the usage of a Neurobasal medium and other factors (such as B27 and cAMP), which are typically employed with primary neuronal cultures [39,53,54,55].

As described here, the currently available protocols do not completely solve the problem of phenotypic heterogeneity, whereby RA administration or the sequential exposure to RA+TPA, and RA+BDNF does not guarantee a purely DAergic phenotype [48,56]. Moreover, several studies reported limitations characterizing these differentiation protocols and their capability to increase the expression of DAergic markers in SH-SY5Y cells [16,17]. This heterogeneity can also introduce variability in experimental results, making it difficult to isolate specific effects and thus to have reliable PD models. For these reasons, many authors preferred not to perform differentiation to avoid additional experimental variables, heterogeneity, and protocol sensitivity introduced by differentiation, especially when the primary aim is toxicological screening rather than neuron-like phenotyping [22,31].

### 3.3. Main Methods for PD Model Induction

To model the PD pathological condition, a diverse set of approaches has been described in the literature, ranging from the use of toxic substances and pesticides to gene transgenesis and gene editing techniques. Among them, the most widely used approach is the one with MPP+ (1-methyl-4-phenylpyridinium), a neurotoxin that mimics the symptoms of the disease by inducing oxidative stress and mitochondrial dysfunction [57]. It damages the inner mitochondrial membrane in DAergic neurons, increasing respiratory dissipation due to the blockage of complex I of the electron transport chain. This induces the production of ROS. When combined with exogenous 6-OHDA, a synthetic catecholaminergic neurotoxin that inhibits mitochondrial complex IV, MPP+, exacerbates oxidative damage and neuronal death in DAergic cells [26]. The MPP+ exposure also causes significant alterations in the cellular processes that are critical for understanding the pathophysiology of PD, impacting the insulin-like growth factor signaling pathway. This leads to a reduction in levels of the insulin receptor, insulin receptor substrate-1, and insulin receptor substrate-2, which play critical roles in neuronal survival and function [58]. The exposure to 6-OHDA also elicits substantial alterations in the cellular processes that are associated with oxidative stress, apoptosis, and the disruption of neurotransmitter metabolism [59]. The quantification of intracellular dopamine and serotonin metabolites provides critical insights into the metabolic dysfunctions induced by this toxin, which mimics those observed in PD [60]. Among pesticides, paraquat and maneb are two frequently employed compounds. The synergistic exposure to these toxins has been demonstrated to significantly impair cellular functions [61] by modulating the protein expression levels, particularly those involved in the Wnt signaling pathway, such as Wnt5a and β-catenin [62]. The dysregulation of these proteins suggests an interference with the critical signaling pathways that are involved in neuronal health maintenance, thereby contributing to the neurodegenerative process [63].

Mechanistically, MPP+ enters DAergic cells and takes advantage of dopamine transporters, such as DAT. Alternatively, MPP+ internalization may occur through pathways involving cationic amino acid transporters expressed in neuronal cells [31]. Similarly, 6-OHDA was reported to utilize a comparable mechanism, as its cytotoxic effects are positively correlated with DAT protein levels [64]. In addition, the paraquat cellular influx into striatal cells has been demonstrated to occur in a sodium-dependent manner, a characteristic hallmark of active transporter-mediated uptake. This suggests a carrier-mediated mechanism rather than simple passive diffusion [65].

Notably, it has been reported that RA-differentiated SH-SY5Y cells exhibit reduced susceptibility to toxin-mediated cell death induced by agents such as 6-OHDA, MPTP, or its metabolite MPP+ [66], while preserving the relevance of this cell line as an in vitro model of PD. Consequently, the selection of the differentiation protocol should be guided by the specific experimental objectives, as distinct differentiation strategies differentially modulate the expression of DAergic markers and the cellular response to specific toxins.

Other approaches are focused on α-Synuclein aggregation, trying to model this key hallmark of PD. They rely on the administration of exogenous α-Synuclein fibrils [67] or the overexpression of α-Synuclein levels to take advantage of the gene transgenesis tools [53,67].

Finally, the modulation of α-Synuclein levels was reported as an approach to improve neuronal deficits typical of the disease, regulating protein aggregation and cellular vulnerability. For instance, mitochondrial ferritin has been reported to downregulate α-Synuclein protein expression, protecting SH-SY5Y cells from oxidative stress [68]. In this context, genome editing technologies represent a promising approach for advancing research on PD using the SH-SY5Y cell model, exhibiting the potential to revolutionize the understanding of the genetic underpinnings of the disease [69]. CRISPR/Cas9 technology is the best choice to facilitate the introduction or correction of mutations, thereby elucidating specific gene functions and their role in disease progression, particularly those associated with α-Synuclein aggregation or mitochondrial dysfunction [68,70,71,72].

## 4. DAergic Phenotype Characterization

### 4.1. Frequency and Temporal Trends of Phenotypic Evaluations in Differentiated Versus Undifferentiated SH-SY5Y Cells

**Figure 3:**
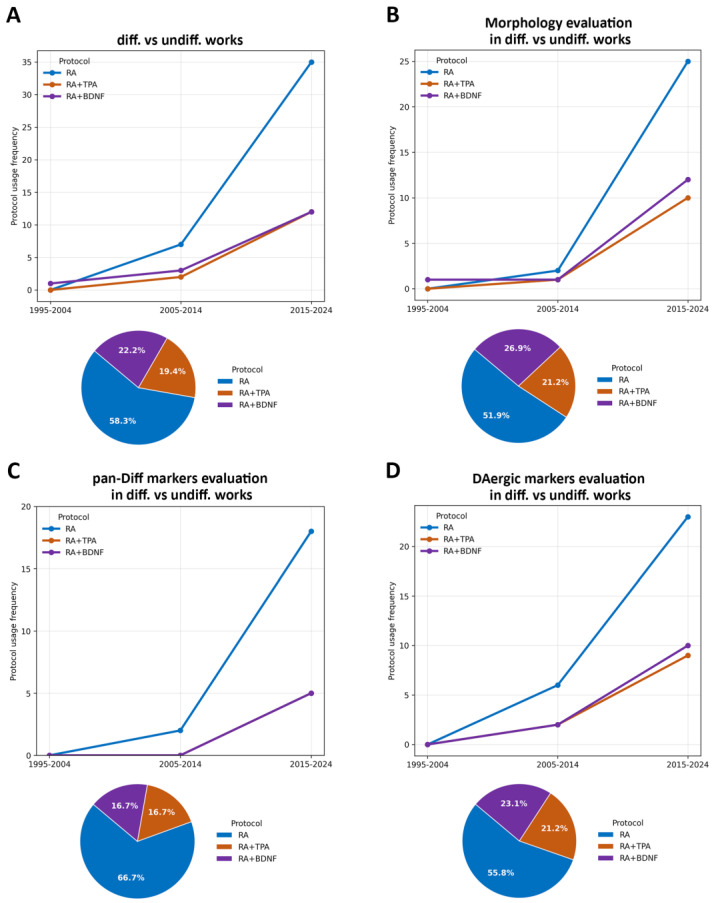
The protocol usage in differentiated vs. undifferentiated SH-SY5Y comparison works, and the protocol for the evaluation of each of the morphological, pan-diff, and DAergic markers. (**A**) On the left is a graph showing the percentage trend in the use of the main differentiation protocols (RA, RA+TPA, and RA+BDNF) reporting the comparison between differentiated and undifferentiated SH-SY5Y cells in three time intervals (1995–2004, 2005–2014, and 2015–2024). On the right is a pie chart showing the percentage of three differentiation protocols over 30 years in studies reporting the comparison between differentiated and undifferentiated SH-SY5Y cells. The same plots were graphed for morphological (**B**), pan-diff marker (**C**), and DAergic marker (**D**) evaluations in differentiated and undifferentiated SH-SY5Y cells comparison works. In panel (**C**), the RA+TPA trend is not separately visible in the line graph because it fully overlaps with the RA+BDNF trend across the analyzed time intervals.

The use of SH-SY5Y cells in PD research critically relies on their ability to acquire a neuronal phenotype that faithfully reproduces the key features of DAergic neurons that are affected in the disease. In the context of PD research, this entails the development of a stable, mature, post-mitotic neuronal phenotype; the acquisition of a DAergic identity; and the expression of functional features that are characteristic of midbrain DAergic neurons. Such properties are not inherent to the undifferentiated neuroblastoma lineage and are largely determined by the differentiation protocol that is employed. To address the question about the capability of the three main differentiation protocols to generate mature DAergic neuronal SH-SY5Y cells, we further screened the 249 studies, selecting only those comparing differentiated versus undifferentiated cells, and reported either morphological assays, markers of neuronal pan-differentiation (pan-diff), or DAergic markers. Finally, we selected a set of markers that have been used at least twice among diverse differentiation protocols (Table S1). Notably, we prioritized the relative expression of markers and morphological changes occurring during the transition from mitotic to post-mitotic neuronal states. Consequently, we excluded studies that evaluated differentiation properties solely in differentiated cells without a direct comparison to undifferentiated controls, since a single measurement is insufficient to adequately characterize a true phenotypic shift or change in status. There were 61 studies that satisfied these requirements, with a total of 72 differentiation protocols. Of these, 42 exclusively used an RA inducer (58.3%), 14 used RA in combination with TPA or TPA alone (19.4%), while 16 used the RA+BDNF protocol (22.2%) (Figure 3A). It is important to note that our analysis evaluated each morphological parameter and molecular marker independently. This approach reflects the common practice in the literature, where these three protocols are frequently analyzed individually. Consequently, at the conclusion of this section, we will discuss the specific studies that have directly compared two or more differentiation protocols.

Initially, we assessed the temporal trends associated with the RA, RA+TPA, and RA+BDNF differentiation protocols regarding their application in evaluating morphology, neuronal pan-diff markers, and DAergic markers. The utilization of all three protocols has shown a progressive increase over time, with the RA protocol being the most frequently represented. Interestingly, most of these evaluations were published within the last decade, indicating a growing research interest in this field (Figure 3B–D, Table S1).

From a morphological perspective, all three protocols successfully induced the SH-SY5Y cells to develop complex neuronal networks, which are characterized by significant increases in neurite length and branching. Morphological assessments were performed in 52 out of 72 of the analyzed protocols (72.2%; Table S1).

### 4.2. Neuronal and DAergi Markers Analysis Across Differentiation Protocols

Subsequently, we focused on the specific markers that were utilized to evaluate differentiation and proliferation properties, as well as DAergic identity. We identified eleven neuronal pan-diff markers across 31 out of 72 analyzed protocols (43.1%, Table S1). These include β-III tubulin (*TUBB3*), which is a canonical neuronal marker that is present in both immature and mature neurons [60,73,74], and several mature neuronal markers, such as neurofilament light chain (*NEFL*), neurofilament heavy chain (*NEFH*), microtubule-associated protein 2 (*MAP2*), RNA binding fox-1 homolog 3 (*RBFOX3*, also known as NeuN), and enolase 2 (*ENO2*) [75]. Furthermore, we examined the synaptic markers, including synaptophysin (*SYP*) and growth-associated protein 43 (*GAP43*), which are expressed at the level of synaptic vesicles and axonal growth cones, respectively [76,77]. Additionally, the phosphorylation of mitogen-activated protein kinase 1 (*p*-MAPK1, also known as *p*-ERK) was included, as it mediates cellular differentiation in response to external stimuli, such as growth factors or oxidative stress [54]. Finally, we evaluated the proliferative markers, whose expression was expected to be downregulated upon differentiation, including the marker of proliferation Ki-67 (*MKI67*), which is an indicator of active cell cycling [78], and nestin (*NES*), which is an intermediate filament protein typically highly expressed in neural stem cells [75].

Among the DAergic markers, eight different genes were evaluated in 51 out of 72 protocols (70.8%, Table S1). Tyrosine hydroxylase (*TH*), which is involved in the conversion of L-tyrosine to L-DOPA and is the rate-limiting step in the biosynthesis of catecholamines, such as dopamine, noradrenaline, and adrenaline [79], was evaluated. Transporters and receptors of dopamine, such as the synaptic vesicular amine transporter (*SLC18A2*, also known as VMAT2), the sodium-dependent dopamine transporter (*SLC6A3*, also known as DAT), and the dopamine receptor D2 and D3 (*DRD2* and *DRD3*) [9], were also evaluated. Then, the transcription of factor paired-like homeodomain 3 (*PITX3*) is crucial for midbrain DAergic neuron differentiation and maintenance [74]. Moreover, we also included two Parkinson-related markers, such as α-Synuclein (*SNCA*), which is expressed at the synaptic level, contributing to synaptic vesicle exocytosis [39], and the Parkinsonism-associated deglycase (*PARK7*, also known as DJ-1), which acts as a protective protein against oxidative stress and helps maintain cellular protein quality [80]. The mutations and alterations of these genes are associated with PD, particularly the aggregation of α-Synuclein in DAergic neurons [81].

**Figure 4:**
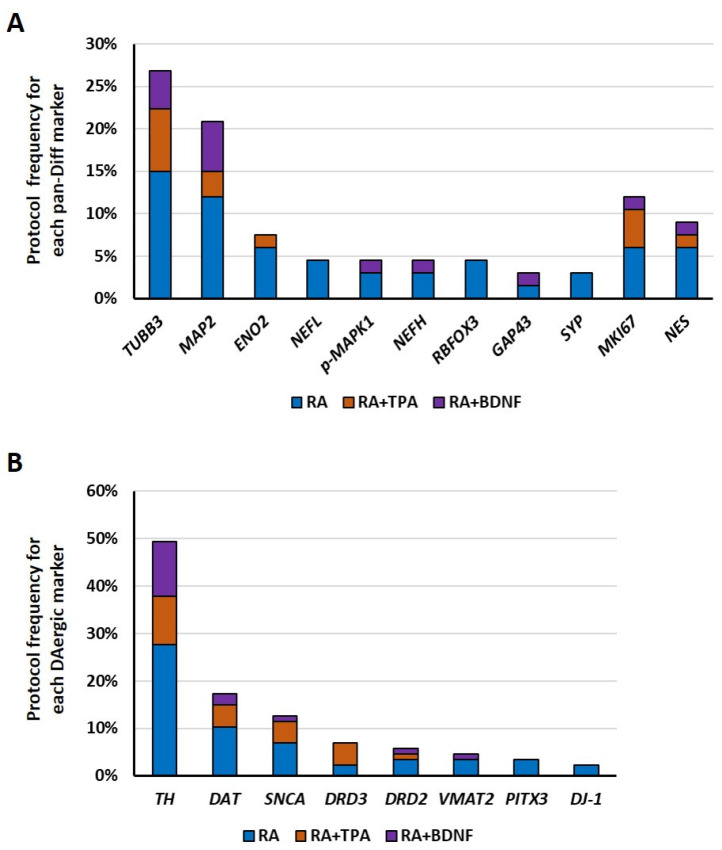
The pan-diff and DAergic markers’ evaluation frequencies per differentiation protocol. The pan-diff markers evaluation frequencies (**A**) and the DAergic markers evaluation frequencies (**B**) in RA, RA+TPA, or RA+BDNF differentiation protocols. The analysis is restricted to studies that compare differentiated to undifferentiated SH-SY5Y cells.

In Figure 4, we illustrated the percentage distribution of pan-diff and DAergic markers among protocols. As shown, the main pan-diff markers were *TUBB3* and *MAP2* genes, along with *MKI67* and *NES* genes as proliferative reporters, a pattern that was consistent across all examined protocols. Among the DAergic markers, *TH* was the most frequently evaluated, whereas *SNCA* was the most commonly studied gene among those associated with PD.

**Figure 5:**
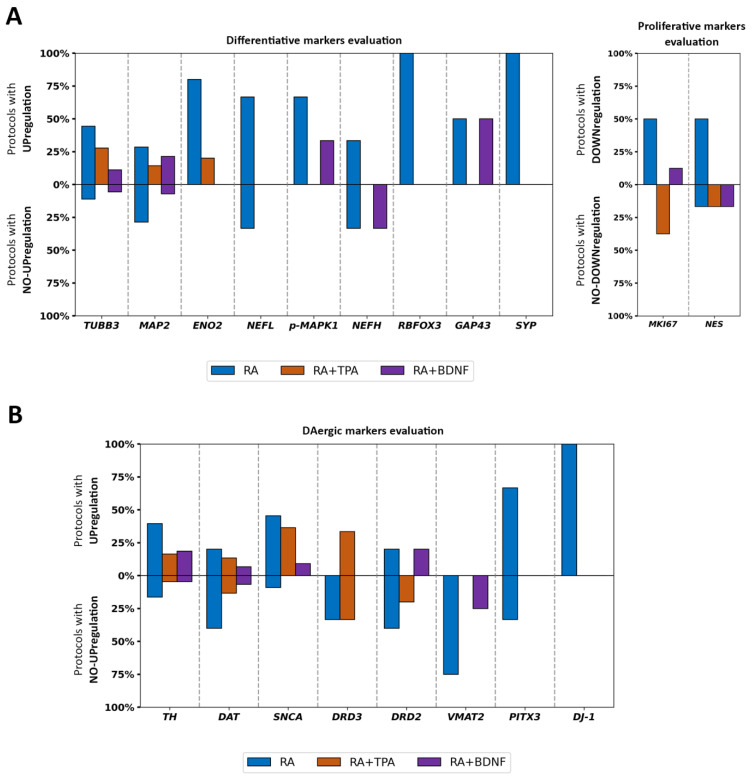
The relative expression levels of pan-diff and DAergic markers in differentiated SH-SY5Y cells. Per each differentiation protocol, these graphs illustrate the frequency of upregulation, in the case of differentiative markers (**A**, **left**) and DAergic markers (**B**), and of downregulation, in the case of proliferative markers (**A**, **right**). Similarly, we reported the frequency of no upregulation/downregulation of each marker along the same y-axis, meaning that no expression differences were observed after the differentiation protocol.

Next, we reported the percentages of protocols that investigated the expression levels of each specific marker, indicating whether they were modified or not in differentiated SH-SY5Y cells compared to undifferentiated cells, relative to the total number of protocols (Figure 5). In most studies, the pan-diff markers increased upon differentiation, independently of the chosen protocol (Figure 5A), except for RA+TPA, which curiously did not decrease the *MKI67* expression levels. Moreover, some studies reported no changes in *TUBB3*, *MAP2*, *NEFL*, *NEFH*, and *NES* expression following the RA or RA+BDNF protocols. Interestingly, *MAP2* was found to be unaltered across the majority of the studies using the RA differentiation protocol. An opposite trend was observed in the studies employing the RA+BDNF protocol, which frequently leads to an upregulation of *MAP2*. These data suggest that all three protocols increase the expression of differentiation markers, with the RA+BDNF protocol being more efficient and potentially inducing greater neuronal maturation. Finally, the RA+TPA protocol appears to only partially inhibit proliferation.

In contrast, regarding the DAergic markers, gene expression levels were frequently found to be similar between undifferentiated and differentiated cells (Figure 5B). This was particularly evident for *DAT*, *DRD3*, and *DRD2* following the RA and RA+TPA protocols, as well as for *VMAT2* under the RA and RA+BDNF conditions. Conversely, *SNCA* was consistently upregulated across all protocols, although it was less frequently represented in the RA+BDNF group, and *DJ-1* was found to be enhanced specifically in the RA protocol. Across protocols, *TH* expression was typically upregulated upon differentiation, although some studies—particularly those using the RA protocol—reported no change in *TH* levels. These data provide quantitative evidence that the expression profile of genes associated with the DAergic phenotype is not entirely consistent across different differentiation protocols, particularly with the use of RA. In contrast, the presence of α-Synuclein appears to be well-characterized regardless of the protocol used.

Consistent results were also obtained when correlating the treatment duration and the fetal bovine serum (FBS) concentration for each inducing agent (Figure S1).

### 4.3. Influence of Differentiation Duration and FBS Concentration in RA Protocols

**Figure 6:**
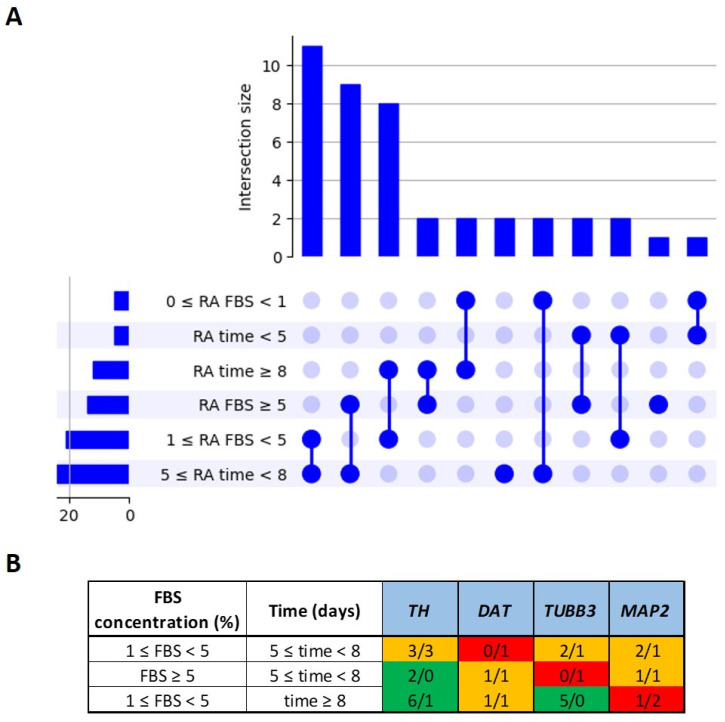
The differentiation days and FBS concentration usage in the RA protocol and marker evaluation. (**A**) The UpSet plot illustrating the frequency of differentiation durations and FBS concentrations used in the RA-induced SH-SY5Y cell protocols (a total of 42 protocols). The plot shows both the number of protocols using individual parameters (bars on the left) and the number of protocols employing specific combinations of differentiation duration and FBS concentration (bars on the top), highlighting the most used conditions across the studies. (**B**) A table showing the three most frequent combinations of differentiation duration and FBS concentration and the ratio of protocols in which the four main markers (*TH*, *DAT*, *TUBB3*, *MAP2*) were either upregulated or unchanged. The green boxes indicate that upregulation was observed in the majority of the protocols (e.g., 6/1), the red boxes indicate that no change predominated (e.g., 0/1), and the orange boxes indicate an inconsistent trend with similar numbers of protocols reporting upregulation and no change (e.g., 1/1).

Focusing on the RA protocol, we investigated whether specific combinations of differentiation duration and FBS concentration could influence the expression of the markers *TUBB3*, *MAP2*, *TH*, and *DAT*. To this end, we defined three ranges of differentiation time (<5 days, 5–8 days, and ≥8 days) and three ranges of FBS concentration (0–1%, 1–5%, and ≥5%). The frequency of each individual parameter and their combinations across the 42 RA-induced SH-SY5Y protocols was visualized using an UpSet plot (Figure 6A), which highlights both the most commonly used conditions and the prevalence of specific combinations. Finally, for each of the three most frequent combinations of differentiation days, exposure, and FBS concentration, we analyzed the expression levels of the *TUBB3*, *MAP2*, *TH*, and *DAT* genes. The markers that were predominantly upregulated were highlighted in green, those showing no change were shown in red, and markers with no consistent trend under the same conditions of differentiation days and FBS concentration were indicated in orange. Unfortunately, the regulation of the *TUBB3*, *MAP2*, *TH*, and *DAT* markers seems to be independent from these two parameters, except for *TH* and *TUBB3* following 1% ≤ FBS < 5% and time ≥ 8 days, which was consistently upregulated (Figure 6B).

### 4.4. Direct Differentiation Protocols Comparison

To consolidate findings on differentiation protocols, we summarized the main results from six studies that directly compared them, since most research investigates these protocols independently.

Marlet et al. [39] compared the RA and RA+TPA protocols, incorporating bone morphogenetic protein 4 (BMP4) to enrich the N-type cell cluster while reducing the S-type populations. Using various combinations of RA and TPA with or without 1% FBS, the authors demonstrated that RA+TPA—particularly in the absence of FBS—significantly increased the neurite length and TUBB3 protein levels compared to RA alone. Notably, with respect to undifferentiated cells, the *MKI67* expression remained unchanged following the RA+TPA protocol, whereas it was reduced by an RA inducer. While TPA enhanced DRD3 protein expression in FBS-free conditions, *TH* levels remained comparable between RA and RA+TPA. The results for α-Synuclein were inconsistent: the Western blot analysis showed decreased levels with TPA compared to RA, yet the mass spectrometry indicated a higher total quantity in TPA-treated cells than in undifferentiated ones. Finally, this study provided unique electrophysiological evidence showing that RA+TPA, in the presence of serum, promotes electrical activity by enhancing action potential amplitude.

El-Habta et al. [82] evaluated *TH* and *DAT* levels over 6 days of treatment with either RA, TPA, or a combination of the two. They found that *TH* mRNA and protein levels increased only with TPA alone, suggesting that RA may suppress this upregulation. In contrast, *DAT* mRNA levels remained stable across all conditions, while DAT protein expression was reduced under RA and RA+TPA protocols but remained unaffected with TPA alone. Furthermore, TPA treatment successfully increased the dopamine levels in the culture medium, corroborating a DAergic phenotype. The comparisons of morphology and differentiation markers were not reported.

Taylor-Whiteley et al. [83] compared the RA and RA+BDNF protocols, measuring the neurite length and a panel of pan-diff and DAergic markers. Specifically, the RA+BDNF treatment led to the most pronounced neurite elongation. Like RA, this protocol maintained low *MKI67* levels, while inducing elevated *NEFH* expression and enhanced intracellular dopamine levels compared to RA. However, no significant over-expression was detected with respect to undifferentiated cells, suggesting that RA inhibits their levels. Although *DRD2* was significantly over-expressed in RA+BDNF compared to RA, other markers such as *DAT*, *MAP2*, *NES*, *TUBB3*, and *VMAT2* showed non-significant increases. In a PD model using exogenous α-Synuclein oligomers, intracellular aggregation was more pronounced in the RA+BDNF protocol, despite a smaller percentage of cells testing positive for α-Synuclein overall.

Avola et al. [84] compared the RA to the RA+TPA protocol. They reported that both protocols are similar regarding morphological and proliferation aspects, increasing the former and decreasing the latter in comparison with undifferentiated cells. However, while *TUBB3* mRNA increased equivalently in both protocols, *MAP2* and *TH* were only over-expressed following RA+TPA treatment, suggesting a more mature DAergic phenotype. Interestingly, the RA+TPA-treated cells exhibited reduced viability and decreased *TH* expression only after chronic (24 h) exposure to MPTP/H2O2, whereas the RA-treated cells were vulnerable to both acute (4 h) and chronic exposure.

Knaryan et al. [85] observed that RA+TPA increased TH protein levels compared to RA. They also cited previous works about the RA+BDNF protocol, which reported the expression of typical cholinergic markers, such as the acetylcholine transferase (*ChAT*) [31]. In addition, they found that RA+TPA-treated cells exposed to mitochondrial toxins (e.g., MPP+ or rotenone) generate reactive oxygen species, whereas RA+BDNF-treated cells exhibit a burst of inflammatory mediators.

Finally, Mastroeni et al. [86] demonstrated that RA+BDNF treatment produced more pronounced neuritic extensions than RA. These cells also exhibited superior DAergic properties, including a higher *TH* and *DAT* expression, as well as an increased dopamine uptake and release.

**Figure 7:**
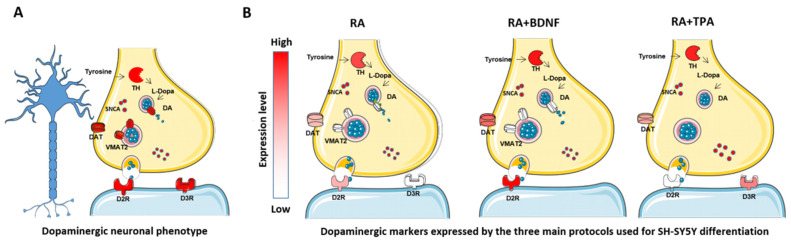
The DAergic markers’ expression undergoing the three differentiation protocols. (**A**) The typical DAergic neuronal phenotype, expressing the main DAergic markers at the synaptic level. (**B**) A schematic representation that summarizes the DAergic markers’ expression data evaluated in the RA, RA+BDNF, and RA+TPA protocols that were included in our screening. Both independent and comparison studies were considered. Specifically, the color intensity follows the proportion between the protocols that reported upregulation of the marker in question and the protocols that reported no upregulation (Table S1). Importantly, these proportions depicted a scenario similar to that evaluated by comparing more protocols together, except for the DAT protein, which was corrected (lowering its intensity) according to comparison studies. If a marker is not depicted, this stands for no presence in our screening for that specific protocol.

In summary, the data described in these two sections confirm the disparate results obtained across various studies regarding the three primary protocols adopted for SH-SY5Y cell differentiation (Figure 7). Interestingly, despite the prevalence of the RA protocol in the literature, the RA+BDNF approach appears to induce the highest expression of DAergic markers compared to other methods. This suggests that the addition of the BDNF may represent the most effective strategy for modeling PD in vitro, as it enhances neuronal maturation and guides the cells toward a DAergic identity. Notably, *VMAT2* expression remained unchanged across all analyzed protocols. Furthermore, no direct comparisons currently exist between the RA+BDNF and RA+TPA protocols, with the latter also lacking a comprehensive molecular characterization. However, studies reporting the expression of cholinergic markers suggest that the RA+BDNF-induced SH-SY5Y cells may lack a strictly unique neurochemical identity [31]. Consequently, further research is required to fully characterize and determine their definitive phenotype.

## 5. Discussion

The SH-SY5Y cell line is extensively utilized as an in vitro model for neurodegenerative disorders. Its capacity to differentiate into various neuronal phenotypes with relative ease and cost-effectiveness, combined with a susceptibility to neurotoxins and neuroprotective agents that are comparable to primary neurons, has made SH-SY5Y cells a preferred tool for dissecting molecular pathogenetic mechanisms and evaluating pharmacological interventions [87]. However, neurodegeneration affects distinct neuronal populations depending on the pathology, for instance, DAergic neurons in PD and cholinergic neurons in AD. Consequently, the selection of an appropriate differentiation protocol is critical to obtaining the desired cellular phenotype. The primary inducing agents that are typically employed include RA, which is either alone or in combination with TPA or BDNF. Interestingly, identical protocols are frequently applied across different disease models, just as diverse protocols are often used to model the same disease [19,22,27,88], frequently assuming reciprocal exchangeability. Furthermore, it is noteworthy that many studies choose to bypass the differentiation process entirely to avoid introducing additional experimental variables [22,31]; however, this approach inherently limits the physiological relevance and investigative power of the model.

This systematic review aims to quantitatively illustrate the impact of differentiation protocols employed in PD research, specifically regarding their capacity to generate a mature DAergic neuronal phenotype. To our knowledge, such a systematic overview is currently missing from the literature. Previous studies have investigated the use of the SH-SY5Y cell line as an in vitro model for PD, addressing it from different yet complementary perspectives [22,27,31]. These works have described the biological features, methodological variability, and principal experimental applications of SH-SY5Y cells. However, they have not performed a quantitative assessment of DAergic neuronal markers across differentiation protocols, relying instead on qualitative analyses. In contrast, our review specifically focuses on phenotypic evaluation by systematically screening studies that directly compare differentiated and undifferentiated SH-SY5Y cells. Furthermore, the most recent systematic review by Xicoy et al. [22] was published in 2017, and the subsequent years up to 2024 have seen a substantial increase in the use of this cellular model (Figure 2 and Figure 3). Accordingly, our analysis captures and integrates this growing body of literature, providing an updated and more comprehensive perspective on the topic. We believe these findings will assist researchers in selecting the most appropriate differentiation protocol, which will be tailored to their specific biological questions. Here, we report evaluations of morphology, differentiation markers, and DAergic markers based on studies comparing differentiated and undifferentiated SH-SY5Y cells.

Our analysis spans the last 30 years and includes a total of 249 studies. Notably, most of these works were published within the last decade (Figure 2B and Figure 3), indicating a significant and growing interest in the use of differentiated SH-SY5Y cells. Among these, 61 studies specifically performed a direct comparison between differentiated and undifferentiated conditions, evaluating morphological parameters and/or molecular markers. It is important to note that some studies included no molecular evaluation; this serves as a warning that the assessment of proper phenotypic differentiation is occasionally omitted or restricted solely to morphological observations (Table S1).

RA was the most frequently used differentiation inducer in the studies included in this analysis. Overall, these studies reported that RA efficiently promotes SH-SY5Y cell differentiation, as indicated by the increased expression of pan-differentiation markers and the concomitant downregulation of proliferative markers. However, the RA-induced cells predominantly express *TUBB3*, a marker of immature neurons, rather than *MAP2*, which is characteristic of mature neurons. This pattern suggests that RA treatment alone does not achieve full neuronal maturation. Regarding the DAergic markers, the landscape is more complex. While RA-induced cells frequently, though not consistently, express the TH protein, this is often not accompanied by a corresponding trend in the DAT, VMAT2, or DRD2/3 protein levels. This observation raises critical considerations regarding phenotypic specificity, as *TH* is actually a marker for a broader catecholaminergic phenotype that includes dopamine, adrenaline, and noradrenaline, rather than being strictly DAergic. Furthermore, the suitability of RA-differentiated SH-SY5Y cells as a robust PD model is called into question by the fact that key DAergic genes are not always upregulated (Figure 5B). This leads to significant mechanistic limitations, particularly concerning the expression of *DAT*, which is essential for toxins like MPP+ to enter the cells and induce the neurotoxicity required to model PD effectively [27,31], yet this transporter is often not upregulated. In contrast, the *SNCA* gene was consistently reported as upregulated during the differentiation across the analyzed studies. Finally, these discrepancies might suggest that specific variables or their combinations, such as the duration of differentiation and FBS concentration, play a critical role in phenotypic specification and marker expression; however, our screening demonstrated that these parameters do not reliably account for the observed variations in the marker regulation (Figure 6).

The sequential administration of RA with TPA or BDNF is notably less represented than RA alone in PD research. Concerning TPA, it can induce the expression of pan-diff genes, although in this case, the *TUBB3* gene remains the predominantly expressed marker. Curiously, the proliferative marker *MKI67* was not found to be downregulated under differentiation, suggesting that these cells may partially continue to proliferate. Regarding the expression of the DAergic markers, the scenario is similar to or slightly improved compared to the RA protocol, even if several genes were not evaluated across the studies. However, *TH* and *DAT* markers were reported to be more highly expressed than in the RA protocol. In summary, our data suggest that despite the general morphological and electrical improvements observed in the SH-SY5Y cells that were differentiated with the RA+TPA protocol, contradictory results regarding proliferative and DAergic markers leave some doubts about its reliability in PD research.

Finally, the BDNF is recognized as a pivotal player in both differentiation and neuronal survival. This is further supported by the higher frequency of studies reporting *MAP2* upregulation during the RA+BDNF differentiation compared to the RA and RA+TPA protocols. Our analysis indicates that the addition of the BDNF promotes high expression levels of DAergic markers, including *TH*, *DAT*, *SNCA*, and *DRD2*, even exceeding the levels observed in other protocols. Nevertheless, other studies that were not included in our primary screening have reported the expression of alternative phenotypic markers [19,31], such as the cholinergic genes *ChAT*, *VAChT* (vesicular acetylcholine transporter), and *AChE* (acetylcholinesterase) [52,89], as well as the noradrenaline release characteristic of a noradrenergic phenotype [25]. Altogether, the integration of our data with the existing evidence suggests specific limitations in the use of RA+BDNF-induced SH-SY5Y cells for PD research, as these cultures may exhibit significant phenotypic heterogeneity.

### Future Perspectives

Overall, our analysis reveals high variability across the marker expression that was induced by diverse differentiation protocols. While each protocol successfully induces characteristic neurite extensions, molecular evaluations often yield contradictory results, with the notable exception of SNCA upregulation. This variability underscores the influence of specific experimental variables on the phenotypic specification of differentiated SH-SY5Y cells.

Consequently, it is essential to verify key biomarkers every time a differentiation protocol is carried out to confirm the proper phenotypic specification. Moreover, to enable more robust discrimination among the differentiation protocols, future studies should prioritize direct comparisons of multiple protocols within the same experimental framework, systematically modulating key variables to quantify their effects on morphological and molecular outcomes and their downstream impact on PD modeling. Moreover, for a better/deeper phenotypic characterization of SH-SY5Y cells, an interesting follow-up study would be to further analyze the literature, considering the studies of SH-SY5Y cells as models of other disorders, such as AD.

Notably, there is a lack of comprehensive transcriptomic data comparing the undifferentiated and differentiated SH-SY5Y cells [40,90], as well as studies comparing different protocols simultaneously. Moreover, further studies are needed to evaluate whether these protocols lead to synapse formation and full morphological maturation. This gap in the literature limits the ability to draw definitive conclusions regarding the capacity of these cells to acquire a bona fide mature DAergic neuronal phenotype.

**Figure 8:**
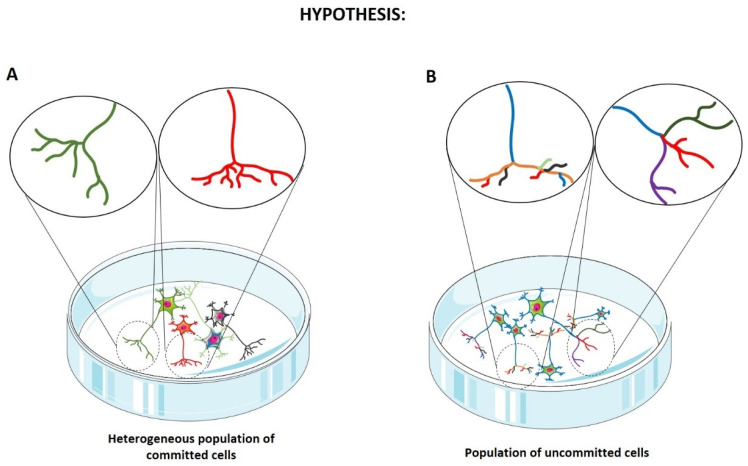
An illustration of the heterogeneity of the differentiated SH-SY5Y cell line in vitro culture. (**A**) The case of a heterogeneous population of committed cells, characterized by different and specific phenotypes. (**B**) The case of a population of uncommitted cells, characterized by undefined phenotypes. Colors are used for illustrative purposes only and do not indicate specific molecular markers. In panel (**A**), different colors represent different committed neuronal phenotypes. In panel (**B**), the cell bodies are shown in the same color to indicate an uncommitted population, whereas the different colors in the neuritic processes indicate mixed or undefined phenotypic synapses.

Importantly, the observation that differentiated SH-SY5Y cells express diverse phenotypic markers alongside the DAergic markers raises two critical hypotheses: either individual cells simultaneously express multiple marker types, defining a population of uncommitted cells, or the phenotypic fate varies between cells, resulting in a heterogeneous population of differently committed cells (Figure 8). To address these possibilities, single-cell RNA sequencing analysis would be highly appropriate, as it allows for the evaluation of the transcriptomic profile of individual cells within a population that is undergoing a specific differentiation protocol. Integrating this approach with gene editing technologies, such as CRISPR/Cas9 [69], would significantly enhance the translational suitability of differentiated SH-SY5Y cells for modeling neurodegenerative diseases, ultimately strengthening their role in preclinical research.

Finally, an area that remains underexplored in the literature concerns the electrophysiological characterization of differentiated SH-SY5Y cells. In our screening, only one study compared the electrical properties of undifferentiated cells with those undergoing differentiation via RA alone or the RA+TPA protocol, demonstrating that the addition of TPA significantly increases the action potential amplitude [39]. The capacity to generate action potentials is fundamental to neurotransmission and serves as a critical hallmark of the mature neurons. While the investigation of pharmacological treatments, neurotoxins, and molecular pathogenetic mechanisms may be conducted in neuron-like cells lacking full electrical maturity, research topics, such as neuronal plasticity, synaptogenesis, and general neurophysiology, cannot be adequately addressed in such models. Consequently, further studies are required to determine which differentiation protocols ensure the acquisition of complete neuronal electrophysiological properties, thereby expanding the utility of SH-SY5Y cells in functional neuroscience.

## 6. Conclusions

This review provides a quantitative assessment of the differentiation protocols employed for SH-SY5Y within PD research. Our analysis highlights that, although the SH-SY5Y model is a well-established system in PD research, marker heterogeneity and the lack of a stable DAergic phenotype remain critical issues. Although the RA treatment resulted in the most frequently used protocol, it showed substantial variability in the expression of DAergic markers. Notably, these limitations appear to be partially improved when RA is combined with BDNF. However, our analysis underscores the need for more standardized and systematic investigations of the SH-SY5Y differentiation protocols to improve the phenotypic consistency and reproducibility in PD research. Future studies should prioritize standardized and reproducible experimental frameworks enabling the direct comparisons of multiple differentiation protocols, combined with rigorous validation of neuronal and DAergic biomarkers. In addition, comprehensive transcriptomic approaches, particularly single-cell RNA sequencing, will be crucial to clarify the extent of cellular heterogeneity and phenotypic specification in differentiated cultures. Finally, further work should determine whether these protocols support synapse formation and full electrophysiological maturation, which are essential features of functional neurons and critical for expanding the translational relevance of SH-SY5Y-based models. By providing a comprehensive overview of the differentiation strategies that are currently employed for the SH-SY5Y cells, this review advances the current understanding of in vitro models in PD research and underscores the critical need for future protocol optimization to improve reproducibility and consistency.

## Data Availability

No new data were created or analyzed in this study. Data sharing is not applicable to this article.

## Supplementary Materials

The following supporting information can be downloaded at https://www.mdpi.com/article/10.3390/ijms27083355/s1.

## Abbreviations

The following abbreviations are used in this manuscript:

6-OHDA	6-hydroxydopamine
AD	Alzheimer’s disease
BDNF	Brain-derived neurotrophic factor
BMP4	Bone morphogenetic protein 4
DA	Dopamine
DAergic	Dopaminergic
DAT	Sodium-dependent dopamine transporter (SLC6A3)
DβH	Dopamine-β-hydroxylase
DJ-1	Parkinsonism-associated deglycase (PARK7)
DRD2	Dopamine receptor D2
DRD3	Dopamine receptor D3
ENO2	Enolase 2
FBS	Fetal bovine serum
GAP43	Growth-associated protein 43
iPSCs	Induced pluripotent stem cells
MAP2	Microtubule-associated protein 2
MKI67	Marker of proliferation Ki-67
MPP+	1-methyl-4-phenylpyridinium
NA	Norepinephrine (noradrenaline)
NEFH	Neurofilament heavy chain
NEFL	Neurofilament light chain
NES	Nestin
NeuN	RNA binding fox-1 homolog 3 (RBFOX3)
Pan-diff	Neuronal pan-differentiation
PD	Parkinson’s disease
PITX3	Paired like homeodomain 3
RA	Retinoic acid
SNCA	α-Synuclein
SYP	Synaptophysin
TH	Tyrosine hydroxylase
TPA	12-O-tetradecanoylphorbol-13-acetate
TUBB3	β-III tubulin
VMAT2	Synaptic vesicular amine transporter (SLC18A2)
