# Supplementary material for: Dopaminergic Identity of SH-SY5Y Cells Across Differentiation Protocols in Parkinson’s Disease Research: A Systematic Review
Source: Int J Mol Sci. 2026 Apr 8;27(8):3355. doi: 10.3390/ijms27083355 (PMC13115675; doi:10.3390/ijms27083355)
Supplement: Supplementary file 1 [file ijms-27-03355-s001.zip › ijms-4187607 - caption.pdf]

Figure S1: Correlation heatmap between differentiation protocol parameters and selected pan-Diff and DAergic markers expression. Pearson correlation coefficients were computed between differentiation protocols defined by inducer exposure timing (days of RA, TPA, or BDNF treatment) and FBS concentrations, and the expression of marker genes associated with DAergic identity, neuronal differentiation, and proliferation (*TH*, *SLC6A3*, *SNCA*, *DRD2*, *DRD3*, *TUBB3*, *MAP2*, *MKI67*, *NES*). The correlation matrix was visualized in Python using Seaborn and Matplotlib libraries, with correlation values annotated within each cell and displayed on a centered cool–warm color scale (blue, negative; red, positive). Blank cells indicate undefined correlations due to missing data or lack of variance.

Table S1: Study screening, data extraction, and primary datasets used for figure generation. The first worksheet, named "Studies retrieved by databases" includes the 514 studies (no duplicated) retrieved from four databases (Pubmed, Web of science, Scopus, Embase) and satisfying criteria selection. For each study it is reported: PubMed Identifier (PMID), Digital Object Identifier (DOI), title, year of publication, publication type, source (database), and criteria of inclusion/exclusion. The second worksheet, named "Screening of included studies" includes the 249 studies that differentiated SH-SY5Y cells via "RA", "RA+TPA", or "RA+BDNF" protocol. For each of them, we specify the type of protocol and a binary indicator for whether the study included a direct comparison between differentiated and undifferentiated conditions. The third worksheet, named "Morph. and markers evaluation" summarizes the subset of studies that performed differentiated-versus-undifferentiated comparisons (n = 72 protocols), detailing induction conditions (inducer(s), exposure time, and FBS percentage). Moreover, it is reported for every protocol the direction of change in outcomes, upregulation (UP), downregulation (DOWN), no change (NS), for pan-Diff differentiation markers (*TUBB3*, *MAP2*, *NEFL*, *ENO2*, *p-MAPK1*, *NEFH*, *RBFOX3*, *SYP*, *GAP43*), proliferation-related markers (*MKI67*, *NES*), DAergic markers (*TH*, *SLC6A3*/DAT, *SNCA*, *DRD3*, *DRD2*, *SLC18A2*/VMAT2, *PITX3*, *PARK7*/DJ-1), and neurite length. Empty cell stands for none information retrieved. Finally, we also included information, if present, about passages number, STR profiling, and mycoplasma test. The fourth worksheet, named "Primary data for Figures" provides the aggregated counts underpinning the corresponding main-text Figures 2, 3, 4, and 5.
