# Supplementary figures and images for: Dopaminergic Identity of SH-SY5Y Cells Across Differentiation Protocols in Parkinson’s Disease Research: A Systematic Review
Source: Int J Mol Sci. 2026 Apr 8;27(8):3355. doi: 10.3390/ijms27083355 (PMC13115675; doi:10.3390/ijms27083355)

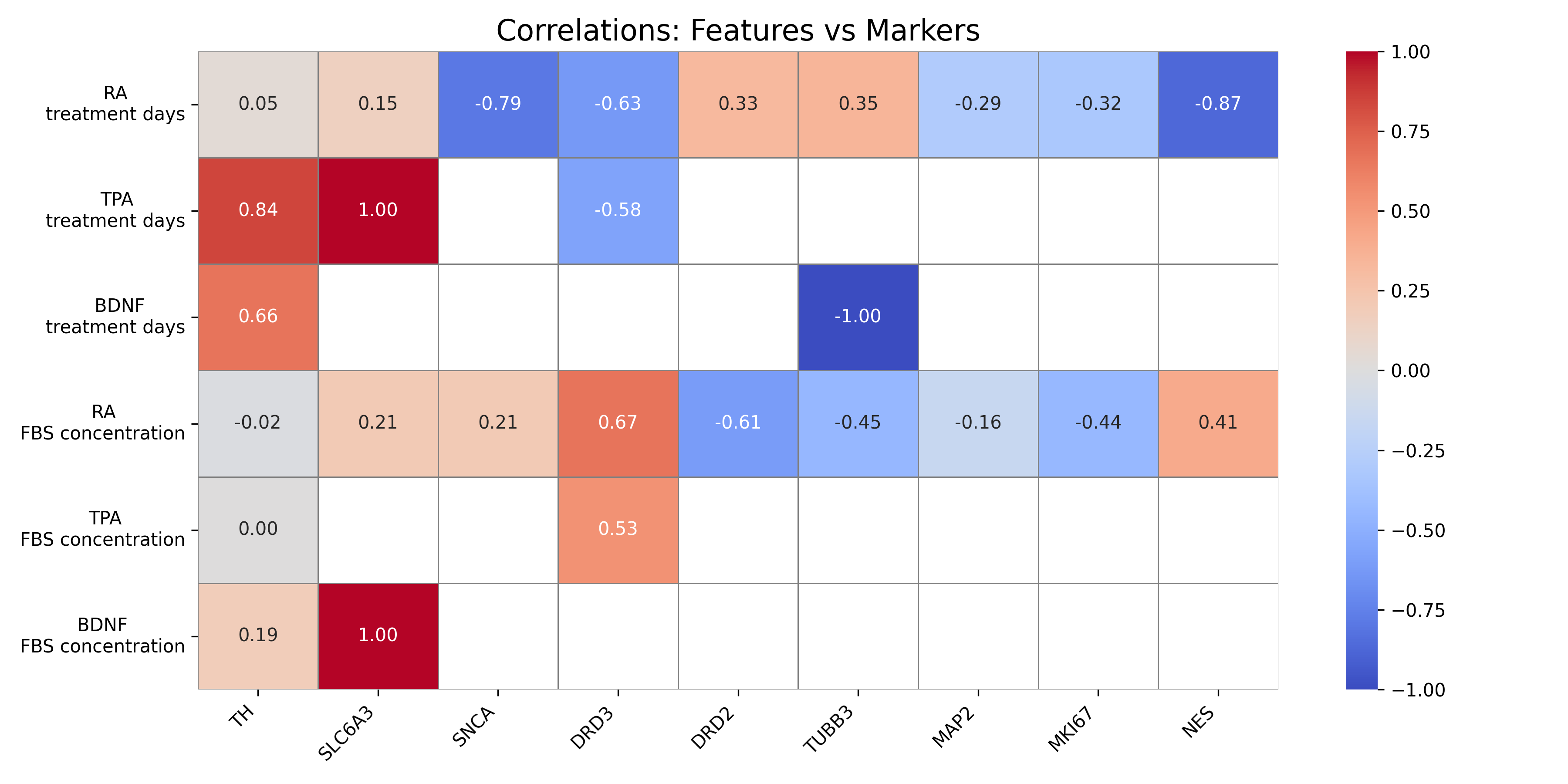

Supplement: Supplementary file 1 [file ijms-27-03355-s001.zip › Figure S1_rev.png]
